# Digital Health, Universal Right, Duty of the State?

**DOI:** 10.5935/abc.20190161

**Published:** 2019-09

**Authors:** Marcelo Antônio Cartaxo Queiroga Lopes, Gláucia Maria Moraes de Oliveira, Luciano Mariz Maia

**Affiliations:** 1 Hospital Alberto Urquiza Wanderley, João Pessoa, PB - Brazil; 2 Universidade Federal do Rio de Janeiro, Rio de Janeiro, RJ - Brazil; 3 Centro de Ciências Jurídicas da Universidade Federal da Paraíba, João Pessoa, PB - Brazil

**Keywords:** Comprehensive Health Care/legislation & jurisprudence, Personal Health Services/trends, Telemedicine, Unified Health System, Health Public Administration

## Introduction

The Brazilian Constitution establishes, in its art. 196,^1^ that “health is a universal right and duty of the State, guaranteed by social and economic policies aimed at reducing the risk of diseases and other ailments, and universal and equal access to actions and services for their promotion and recovery”.

In addition, article 198,^1^ states that “public health actions and services are part of a regionalized and hierarchical network and constitute a single system, organized with the following guidelines: I - decentralization, with a single direction in each sphere of government; II - integral care, with priority for preventive actions, without loss to the care services; III - community participation”.

From a systematic reading of these two constitutional provisions, it is possible to list the basic elements of the implementation of the right to health by the public authorities: universal and equal access, as well as integral care. Integral health care determines that “the duty of the State cannot be limited, mitigated or divided, since health as an individual, collective and development asset presupposes a complete approach to care” and providing integral care “means nothing more than privileging life to the detriment of the administration's budgetary interests - the so-called secondary public interest”.^2^

Within this context, the Unified Health System (SUS) was designed to be the mechanism by which universal and equal access, as well as integral care, should be implemented. SUS must act according to these guidelines, not being able to impose any restrictions specifically directed to a particular group or class, nor can it privilege the administration’s budgetary interests to the detriment of the right to life.

As observed by Resende^,3^ “the concept of health as a fundamental right in the international normative framework has been extended over the years to include, in addition to the negative idea of absence of disease, positive content related to the improvement in quality of life and wellbeing”. According to the Bangkok Charter^4^ for Health Promotion in a Globalized World, drafted at the VI Global Conference on Health Promotion in 2005, “The United Nations recognizes that the enjoyment of the highest attainable standard of health is one of the fundamental rights of every human being without discrimination. Health promotion is based on this critical human right and offers a positive and inclusive concept of health as a determinant of the quality of life and encompassing mental and spiritual well-being”.

Access to health is a social right, guaranteed in Article 6 of the Constitution,^1^ in accordance with the dignity of the human person, which is the basis of the democratic rule of law. The Constitution, said to be citizen-oriented, inaugurated a new legal order in the country that promotes the inclusion of millions of Brazilians who were excluded from any kind of healthcare.

Putting it into perspective, at the beginning of the 20^th^century, only those who integrated welfare funds had access to the health system. Even with the unification of the Institutes of Social Security Assistance, the so-called IAPs, and the creation of the National Institute of Social Security (INPS), there was still the exclusion of non-participants - non-taxpayers, a true legion of indigents.

SUS, observing the federative organization of the Brazilian State, was conceptually conceived as a solution, but after three decades, chronic problems of financing and management persist, jamming the gears of the world's largest system of universal access to healthcare, hampering the achievement of its original objectives. The gigantism of Brazil and the heterogeneity of the different regions impose the need for efficient management that can be capable of promoting, within the priorities of the State, the convenient allocative justice. Only with the adoption of consequent public policies and its capillarity in the whole country will it be possible to change the panorama of public health in Brazil.

In recent decades, as a consequence of the success of public policies, life expectancy has increased, and we are currently experiencing a real demographic transition. The growth in the number of elderly people is exponential, and it is estimated that this social segment will represent 25% of the Brazilian population in 20 years. The impact on social security is a challenge for the State, and requires increased attention, with the adoption of sustainable public policies, especially in the area of health.^4^

Non-communicable chronic degenerative diseases (NCDs) are responsible for more than 30% of global mortality and this context will be aggravated by the aging and sickness of the current population. As a matter of fact, there is no way to ignore that offering the resources needed for the expansion of health care, especially for the growing prevalence of NCDs, mainly in remote areas of a country such as Brazil, is a complex task. It will be a challenge, to be overcome, to bring health professionals - notably specialists - to the most distant corners of the country.^4^

On the other hand, there is a constant tension between the medical class and health authorities about the possible alternatives for the supply of doctors to society. This theme took on an epic battle line on the occasion of the sanction of Law 12.871, dated October 22, 2013,^5^ which instituted the “*Programa Mais Médicos*” (More Doctors Program) in Brazil. The offer of doctors in the country, since the institution of this Program, has increased, mainly due to the increase in the number of medical schools opened in the country. However, the proportion of doctors per 100,000 inhabitants is still below the average of the countries in the Organization for Economic Cooperation and Development (OECD). It should be added that the distribution of these physicians is heterogeneous, aggravating this scenario.^6^ In this sense, the use of information and communication technologies, through a Telemedicine network, could contribute to the universality and integrality of the health system, in line with the constitutional decree.

### Telemedicine as a tool to expand universal access to health

The remarkable advancement of information and communication technologies and their application in medicine have enabled a secure transmission of data, facilitating the interaction of health professionals, opening a door for the democratization of access to medical knowledge, and strengthening collaboration among the various levels of healthcare.

Telemedicine can be conceptualized as an organized and efficient way of practicing distance medicine for the purposes of informing, diagnosing and treating individuals, alone or in groups, based on data, documents or other reliable information transmitted by means of telecommunications. Currently, the use of telemedicine has been increasing, ranging from consultations (teleconsultation), diagnosis (telediagnosis) to complex robotic surgeries (tele-surgery). All these advancements, which project the broader concept of digital health, must take place preserving the millennial postulates of medical art, always focused on the patient’s best interests.^7^

Digital health interventions are not a substitute for the health systems already in place, as there are still significant limitations to what digital health is capable of addressing. However, the judicious use of this technology can contribute to improvements in health care, as long as it is based on the evaluation of its benefits, damages, acceptability and viability.^7^

When approaching the intricate subject of health-guaranteeing regulations, as a fundamental right, and of the application of information and communication technologies to promote the practice of distance medicine, the basis of Telemedicine, there is the need to defend the preservation of the patients' privacy. The inviolability of private life, such as the right to health, is also an inseparable part of the concept of the dignity of the human person.^1^ Therefore, security in data transmission is imperative for the implementation of any Telemedicine program. The millennial principle of medical secrecy, valid since Hippocrates, is also a constitutional imposition.

The legal framework applicable to Telemedicine in Brazil is comprehensive, involving from the sanitary legislation to the recent internet regulatory framework to ensure its regular practice. Currently, international protocols ensure the secure transmission of data: the Health Insurance Portability and Accountability Act (HIPPA)^7^ contains a set of standards that ensure the security of the data transported and of those responsible for its transmission.

### Regulation of Telemedicine by the Brazilian Federal Council of Medicine

The Brazilian Federal Council of Medicine (CFM), an autarchic body established by Law 3.268, dated September 30, 1957,^8^ is responsible for supervising professional ethics throughout the Republic and, at the same time, judging and disciplining the medical profession through supervisory and regulatory action. With a focus on these attributions, CFM is responsible for regulating the participation of doctors in activities related to the employment of Telemedicine throughout the country’s territory.

Law 12.842,^9^ dated July 10, 2013, the Medical Act, which provides for the practice of medicine, ratifies that the new medical procedures and therapies for regular use in Brazil must necessarily be evaluated by the Brazilian Federal Medical Council regarding safety, efficiency, convenience, and benefits to the patient. Telemedicine, with a myriad of employment possibilities in prevention, diagnosis, treatment and rehabilitation, as well as in health promotion, undoubtedly falls within and can be validated by CFM. Because of its innovative character, it brings a bioethical conflict potential, which imposes a zetetic - investigative - analysis of its principles, due to the clash between traditional ethics, which permeates the face-to-face relationship between doctors and patients, and the new frontier opened by the progress of information and communication technology.

The Brazilian Federal Medical Council, through Resolution CFM 1.643/2002,^10^ provided for Telemedicine, defining it “as the exercise of Medicine through the use of interactive audiovisual and data communication methodologies, with the objective of health assistance, education and research”. This regulation requires the use of appropriate technology and observance of the CFM’s technical standards regarding data storage, handling and transmission, confidentiality, privacy and guarantee of professional secrecy. The role of doctors who participate in the professional act at a distance is restricted to emergencies, or when requested by the doctor in charge of administering in-person care.

In 2014, the CFM again expressed its views on the subject, through Resolution CFM 2.107/2014,^11^ to discipline the use of Teleradiology. This Resolution updated the previous standard, published in 2009. The development of technology and the democratization of access to cellular telephony pluralized the development of applications dedicated to digital health. Current possibilities of employment of Telemedicine include several services, which include: (a) Teleconsulting, Teleinterconsulting, Telediagnosis, Tele-orientation, Telemonitoring, Tele-surgery and Medical Tele-Screening. Although part of these services is not explicitly regulated by the CFM, there is an offer by specialized companies, especially in the scope of supplementary health, with the imperative need by such companies of their own administrative act.

In this sense, the CFM issued Resolution CFM 2.227/2018,^12^ published in the Brazilian Federal Official Gazette on February 6, 2019, to update the current discipline. The resolution aimed to guarantee security to the provision of medical services mediated by information and communication technologies in Brazil. There is no doubt of the need to update the regulatory framework that disciplines the participation of doctors in the so-called Telemedicine. This measure legitimizes the doctor-patient relationship in the field of digital health. However, there was an avalanche of questions from the medical category about the form and merit of this standard.

In their article *Window to the future or door to chaos?*, Lopes et al.^13^ discussed several aspects related to the legality and timeliness of Resolution CFM 2.227/2018, and the allowing of Teleconsultations was the most challenging question, precisely due to the flexibility of prescription without the direct examination of the patient, a conduct that is prohibited by the Brazilian Code of Medical Ethics.* For the authors, the regulation of CFM “should therefore represent a step forward, not a setback. Broadening access to public health is a common desire of all doctors. The major challenge of Resolution CFM 2.227/2018 would be having the effectiveness and applicability to move forward in the field of justice and deliberative ethics”.

The avalanche of corporate questions from the medical category, among other reasons, motivated the revocation of this Resolution by CFM.^11^ Thus, the use of telemedicine by doctors in Brazil must occur according to the provisions of Resolution CFM 1.643/2002.^10^ It is noteworthy that there were problems in communication regarding the contents of the norm, generating an intense reaction from doctors in relation to the merit of the regulation.

In addition, as discussed by Lopes et al.,^13^ CFM could not delegate exclusively to specialty societies the prerogative of developing guidelines on Telediagnosis. It is important to emphasize that Law 12.401, dated April 28, 2011,^9^ defines that the elaboration of clinical protocols and therapeutic guidelines within SUS is a jurisdiction of the National Commission for the Incorporation of Technologies in SUS (Conitec). Therefore, CFM could not, on the basis of a normative resolution, exclude those who have legal jurisdiction to elaborate guidelines within the Brazilian health system, delegating this attribution exclusively to private entities, even if conditioned to their approval. Hence, whether in relation to Robotic Telesurgery or Telediagnosis, the revoked Resolution could be improved.

### Difficulties for the implementation of digital health as a duty of the State

In this sense, we could imagine Telemedicine as a useful complementary tool to allow fair access to health for all Brazilians, regardless of ethnicity, gender, socioeconomic status and location in the national territory. It could be assumed, especially if we consider the continental dimension of Brazil, that populations living in remote areas would benefit from the State’s investment in the dissemination of digital health.

According to the Brazilian Institute of Geography and Statistics (IBGE),^14^ about 65% of the municipalities located in remote areas are located in the North and Midwest regions of the country. On the other hand, the study Demografia Médica no Brasil (Medical Demography in Brazil, 2018)^6^ reported significant inequality in the distribution of doctors, who are predominately located in the large urban agglomerations of the South and Southeast Regions, which also concentrates the largest number of specialists, with he North and Northeast Regions having a lower medical/inhabitant density. If we also look at the issue from the perspective of care, through the National Register of Health Establishments (CNES)^15^ of the Brazilian Ministry of Health, we can observe a greater concentration of medical activity in the Southeast and South Regions. It is also important to mention that there is a lower concentration of networks linked by fiber optics in municipalities in the North Region, and that mobile cellular telephone coverage for the Brazilian population is between 98 and 99%, with a higher concentration in the urban centers of the Southeast and South regions.^16,17^

It is understood that, although the demand for medical services in remote areas is an opportunity, the provision of Telemedicine services to these areas presents a great implementation challenge similar to the universal access to traditional health services. The expansion of Telemedicine would have to be preceded by improved digital technology infrastructure.

On the other hand, through the International Telecommunication Union (ITU),^18^ the United Nations has been working with the World Health Organization (WHO) to stimulate the reduction of the global digital divide, with the e-health strategy, focused on digital health, via Telemedicine.

Investments in digital health have generated a number of WHO publications. Examples are the Digital Health Atlas,^19^ a virtual global repository to support governments in monitoring and coordinating digital investments, BeHe@lthy, BeMobile (BHBM),^20^ for prevention and control of NCDs, mHealth Assessment and Planning for Scale (MAPS), a manual for monitoring and evaluation of digital health,^21^ aimed at strengthening the research and implementation of digital health, and the first WHO Digital Health Interventions Guideline.^22^ The latter document^22^ suggests that most of the available scientific evidence on the benefits of implementing global digital health is still not robust, and that there are numerous gaps for large-scale use, albeit in a complementary way to traditional methods. The WHO recommends that a planned process should take place, including: the feasibility of network coverage for access to remote locations, the construction of the legal framework for its implementation, the budget impact and the cost-effectiveness evaluation of each stage of the project's implementation, with the elaboration of indicators of the clinical continuum of applicability for the safety of users.

### Telemedicine to reduce inequalities in the approach to NCDs

Telemedicine, if applied in its broad context, could allow access and equity, offering quality services with supposed cost-effectiveness, especially considering the increase in the prevalence and mortality of NCDs, of which cardiovascular diseases (CVD) are its main component.

There were 55.9 million deaths worldwide in 2017 in an estimated world population of 7.64 billion people.^23^ Of these, 70% were due to NCDs, and are expected to continue to increase in the coming decades, especially in low- and middle-income countries, even though all-cause mortality has decreased, notably due to the reduction of infant and child mortality of children under 5 years, stabilization of mortality from 5 years to 49 years and increase in life expectancy.^23^ The increase in NCDs is expected with the aging of the population, with the control of communicable diseases and with the increase of premature mortality in individuals from 30 to 70 years of age.^24^

In Brazil, NCDs were responsible for about 60% of deaths in 2017, according to the Department of Informatics of the Unified Health System (DATASUS),^25^ noting that this group of diseases shares the same risk factors and social determinants. The severity of the issue is large enough to set targets for 30% reduction of premature mortality by NCDs as part of the Sustainable Development Goals (SDG) for 2030.^26,27^ It is also believed that one-third of the populations of the Americas do not have access to health care, and that an additional 800,000 health workers would be needed to meet the demands of health systems in the region.^28^

The combined approach to NCDs and their risk factors was considered a cost-effective package by WHO, requiring investment of USD 1 per capita in low-income countries, USD 1.5 in low-middle-income countries, and USD 3 in average income countries, underscoring the importance of joint study of NCDs.^29^ According to the WHO Director-General, Dr. Tedros Adhanom Ghebreyesus, “leveraging the power of digital technologies will be essential to achieving the Global Sustainable Development Goals, including universal health coverage, and that such technologies are no longer a luxury, but a necessity”. He also suggests that we make sure that innovation and technology alleviate inequalities, and that countries must be guided by evidence to establish harmonized digital systems, and not be seduced by the novelties.^30^

The World Health Assembly resolution on Digital Health, unanimously adopted by WHO Members in May 2018, demonstrated collective recognition of the value of digital technologies in contributing to the advancement of universal health coverage, with an emphasis on NCDs, and recommended that health ministries evaluate the use of digital technologies dedicated to health, prioritizing development, evaluation, implementation and increased use of these technologies, as well as guiding their standardization, including through the promotion of digital health interventions. However, it was pointed out that, in order to reduce health inequalities, a rigorous evaluation of eHealth strategies would be necessary in order to generate evidence and promote the appropriate integration of the use of these technologies.^18^

### Recommendations of the Telemedicine Directive of the Brazilian Society of Cardiology for cardiovascular health

To guide the practice of Telemedicine in CVD, Lopes et al. developed the Telemedicine Directive of the Brazilian Society of Cardiology,^31^ with the objective of discussing legal and ethical support, technical conditions and priority for implementation, cost-effectiveness and budget impact for the use of Telemedicine for the cardiovascular health of the Brazilian population.

It was found that there is space for Telemedicine initiatives as a specialist matrix support for general practitioners and family health doctors in basic health units in remote areas of the Brazilian territory, especially with regard to diagnostic methods, avoiding unnecessary displacements with additional burden to the health system. The clinical and economic results obtained with public policies focused on digital health in Brazil suggest that technologies that allow patient monitoring (telemonitoring) and the issuance of remote reports (Telediagnosis) applied to cardiology can be cost-effective, with an acceptable impact on the public budget. However, the set of scientific evidence in Brazil is still limited, given the small number of patients involved, to infer that the application in subgroups of clinical interest should be generalized.

The benefits of this technology could be equally applicable to supplemental health in Brazil, even in the face of a diverse regulatory framework, and of the appropriate coverage of face-to-face social assistance. It should be noted that the majority of the beneficiaries of supplementary health reside in larger centers, where the ratio of doctors/specialists per inhabitant is appropriate, and consultations in person are a legal imposition.

Telemedicine may be an important incremental tool in supplemental health, provided there is additional regulation for its implementation. Among the possible measures to extend the scope of Telemedicine in supplementary health, as already exists in American Medicare, would be the inclusion of technologies, with scientific and legal basis, in the Health Procedures and Events List of the National Supplementary Health Agency, since coverage would be mandatory, providing equity and legal certainty.

It is recommended that the bases established by the Brazilian Code of Medical Ethics be maintained in the Telemedicine and Telecardiology procedures. Telemedicine should be considered an additional tool for the face-to-face physician-patient relationship, without ever replacing it.

## Conclusion

Telemedicine as a means of increasing universal and integral access to health, backed by solid evidence, attested by the scientific community, within the budgetary capacity of the Brazilian State, expressed in legitimate public policies, integrates the existential minimum of each Brazilian citizen. It is, therefore, a universal right and duty of the State, and must be guaranteed through social and economic policies in force in the country. The remarkable advance of information and communication technologies and their application in health must be a constant focus of attention of the public authorities, being an instrument of equity and fostering the dignity of the human person. The use of technology in medicine emphasizes the duty of due care to preserve patients’ privacy and the transcendent values that underlie the practice of Medicine.
